# Large Language Model Symptom Identification From Clinical Text: Multicenter Study

**DOI:** 10.2196/72984

**Published:** 2025-07-31

**Authors:** Andrew J McMurry, Dylan Phelan, Brian E Dixon, Alon Geva, Daniel Gottlieb, James R Jones, Michael Terry, David E Taylor, Hannah Callaway, Sneha Manoharan, Timothy Miller, Karen L Olson, Kenneth D Mandl

**Affiliations:** 1 Computational Health Informatics Program, Boston Children's Hospital, 401 Park Drive, LM5506, Mail Stop BCH3187, Boston, MA, 02215, United States, 1 617-355-4145; 2 Department of Pediatrics, Harvard Medical School, Boston, MA, United States; 3 Department of Health Policy and Management, Fairbanks School of Public Health, Indiana University, Indianapolis, IN, United States; 4 Center for Biomedical Informatics, Regenstrief Institute, Indianapolis, IN, United States; 5 Department of Anesthesia, Harvard Medical School, Boston, MA, United States; 6 Department of Biomedical Informatics, Harvard Medical School, Boston, MA, United States

**Keywords:** natural language processing, artificial intelligence, large language models, symptom recognition, clinical text mining, medical informatics, infectious disease surveillance, epidemiologic methods, emergency medical services, electronic health records

## Abstract

**Background:**

Recognizing patient symptoms is fundamental to medicine, research, and public health. However, symptoms are often underreported in coded formats even though they are routinely documented in physician notes. Large language models (LLMs), noted for their generalizability, could help bridge this gap by mimicking the role of human expert chart reviewers for symptom identification.

**Objective:**

The primary objective of this multisite study was to measure the accurate identification of infectious respiratory disease symptoms using LLMs instructed to follow chart review guidelines. The secondary objective was to evaluate LLM generalizability in multisite settings without the need for site-specific training, fine-tuning, or customization.

**Methods:**

Four LLMs were evaluated: GPT-4, GPT-3.5, Llama2 70B, and Mixtral 8×7B. LLM prompts were instructed to take on the role of chart reviewers and follow symptom annotation guidelines when assessing physician notes. Ground truth labels for each note were annotated by subject matter experts. Optimal LLM prompting strategies were selected using a development corpus of 103 notes from the emergency department at Boston Children’s Hospital. The performance of each LLM was measured using a test corpus with 202 notes from Boston Children’s Hospital. The performance of an *International Classification of Diseases, Tenth Revision* (*ICD-10*)–based method was also measured as a baseline. Generalizability of the most performant LLM was then measured in a validation corpus of 308 notes from 21 emergency departments in the Indiana Health Information Exchange.

**Results:**

Symptom identification accuracy was superior for every LLM tested for each infectious disease symptom compared to an *ICD-10*–based method (*F*_1_-score=45.1%). GPT-4 was the highest scoring (*F*_1_-score=91.4%; *P*<.001) and was significantly better than the *ICD-10*–based method, followed by GPT-3.5 (*F*_1_-score=90.0%; *P*<.001), Llama2 (*F*_1_-score=81.7%; *P*<.001), and Mixtral (*F*_1_-score=83.5%; *P*<.001). For the validation corpus, performance of the *ICD-10*–based method decreased (*F*_1_-score=26.9%), while GPT-4 increased (*F*_1_-score=94.0%), demonstrating better generalizability using GPT-4 (*P*<.001).

**Conclusions:**

LLMs significantly outperformed an *ICD-10*–based method for respiratory symptom identification in emergency department electronic health records. GPT-4 demonstrated the highest accuracy and generalizability, suggesting that LLMs may augment or replace traditional approaches. LLMs can be instructed to mimic human chart reviewers with high accuracy. Future work should assess broader symptom types and health care settings.

## Introduction

To practice medicine, accurate identification and interpretation of symptoms are paramount. Symptoms are primary indicators of patient health, underpinning diagnostic processes [1] and choice of therapeutic interventions [2]. Identifying symptoms is also fundamental to public health [3,4], medication safety [5,6], clinical research [7,8], and clinical trials [9-13]. Though symptoms are routinely documented in physician notes, coded formats such as the *International Classification of Diseases, Tenth Revision* (*ICD-10*) [14] often underreport patient symptoms [4,15-18]. The gap between medical coding practices and richer phenotyping has motivated many efforts to develop natural language processing (NLP) of physician notes [17].

Traditional NLP methods for symptom identification [15,18,19] typically target specific note sections [20,21] such as the chief complaint [19,22-24] and often struggle to interpret if or when symptoms are positive [25-27]. The context [18,21,28] surrounding infectious respiratory diseases includes symptoms pertaining to acute infections, noninfectious conditions, treatment side effects [6], indications for treatment, or patient instructions (eg, “Use albuterol inhaler as needed for difficulty breathing”).

Large language models (LLMs) hold potential to overcome such limitations [29,30]. As LLMs are derived from population scale examples, they may better infer symptoms from internet text such as articles about symptom checklists [1,31], disease progression [32], and medical decision-making [2]. Unlike traditional clinical NLP models, LLMs are not trained to any specific domain, which means that LLMs should be more generalizable to documentation variation across health care locations and may not require site-specific training [20,33] to achieve state-of-the-art accuracy.

We sought to measure the accuracy of LLMs for symptom identification, with a focus on infectious respiratory disease symptoms [4]. The code and results are available free of charge with the Apache open-source license 2.0 [34].

## Methods

### Study Design

This is a multisite retrospective study of infectious respiratory disease symptoms documented in electronic health records. Ground truth symptom labels were annotated by human expert chart reviewers. Two symptom identification methods were compared to ground truth labels: (1) an *ICD-10*–based method using coded data and (2) an LLM-based method using unstructured emergency department (ED) notes. LLM prompting strategies were developed for Llama 2 70B Chat [35], Mistral AI Mixtral 8×7B Instruct [36], GPT-3.5 turbo (version 0125) [37] and GPT-4 turbo (version 0125) [37]. The selection of LLMs at the time of experimentation represented the state of the art available in our Health Insurance Portability and Accountability Act (HIPAA)–authorized environments.

### Setting

Boston Children’s Hospital (BCH), a large Northeastern urban pediatric academic medical center, and the Indiana Health Information Exchange (IHIE) [38,39], a Midwestern statewide health information exchange network, were the study sites. Notes from BCH ED patients (aged 21 years and younger) and from IHIE ED patients (any age) with a COVID-19 diagnosis between March 1, 2020, and May 31, 2022, were eligible for inclusion into the study corpus.

### Study Corpus

A study corpus of 613 notes was selected to ensure that it contained examples of rare symptoms. Apache cTAKES [40] was used to first identify positive symptoms in each note. At BCH, notes were then selected to include at least 30 positive examples for each of the 11 symptoms, as well as notes with no positive symptoms. These were used for a development corpus (103 notes) to select optimal strategies for each LLM, and a test corpus (202 notes) to measure accuracy. At IHIE, a validation corpus (308 notes) was randomly selected from a larger sample of 300 positive notes for each symptom and used to assess multisite generalizability in a setting comprising many health care locations.

### Ground Truth

Three BCH experts collaboratively defined inclusion and exclusion criteria for symptom annotation guidelines [4]. They performed iterative cycles of independent chart review, collaborative adjudication of disagreements, and collaborative refinement of symptom annotation guidelines until a consensus was reached. Expert pairs reviewed notes from their own site. Interrater reliability was assessed with the kappa statistic [41,42] (overall mean 0.96, SD 0.07; details in Multimedia Appendix 1).

### Measures

Eleven symptoms related to infectious respiratory disease were measured: congestion or runny nose, cough, diarrhea, dyspnea (shortness of breath), fatigue, fever or chills, headache, loss of taste or smell, muscle or body aches, nausea or vomiting, and sore throat.

*F*_1_-scores, precision, and recall were calculated for each symptom and for all symptoms combined [42]. Micro *F*_1_-scores were used, rather than macro *F*_1_-scores, to allow for stronger competition from *ICD-10*–based metrics, which were quite poor for some symptoms. McNemar tests were used to evaluate LLM versus *ICD-10*–based performance. With an overall α of .05, a Bonferroni adjustment for 12 comparisons (11 symptoms plus no symptoms) set the threshold at *P*<.0042.

### Comparator

*ICD-10* codelists (Multimedia Appendix 2) [4] for each symptom were compiled by 3 experts at BCH using online resources [43,44]. The panel collaboratively reviewed whether each candidate code met the inclusion or exclusion criteria defined in the symptom annotation guidelines. *ICD-10* codes recorded at the time of ED discharge were matched against the final symptom codelists.

### Prompt Engineering

**Figure 1.**
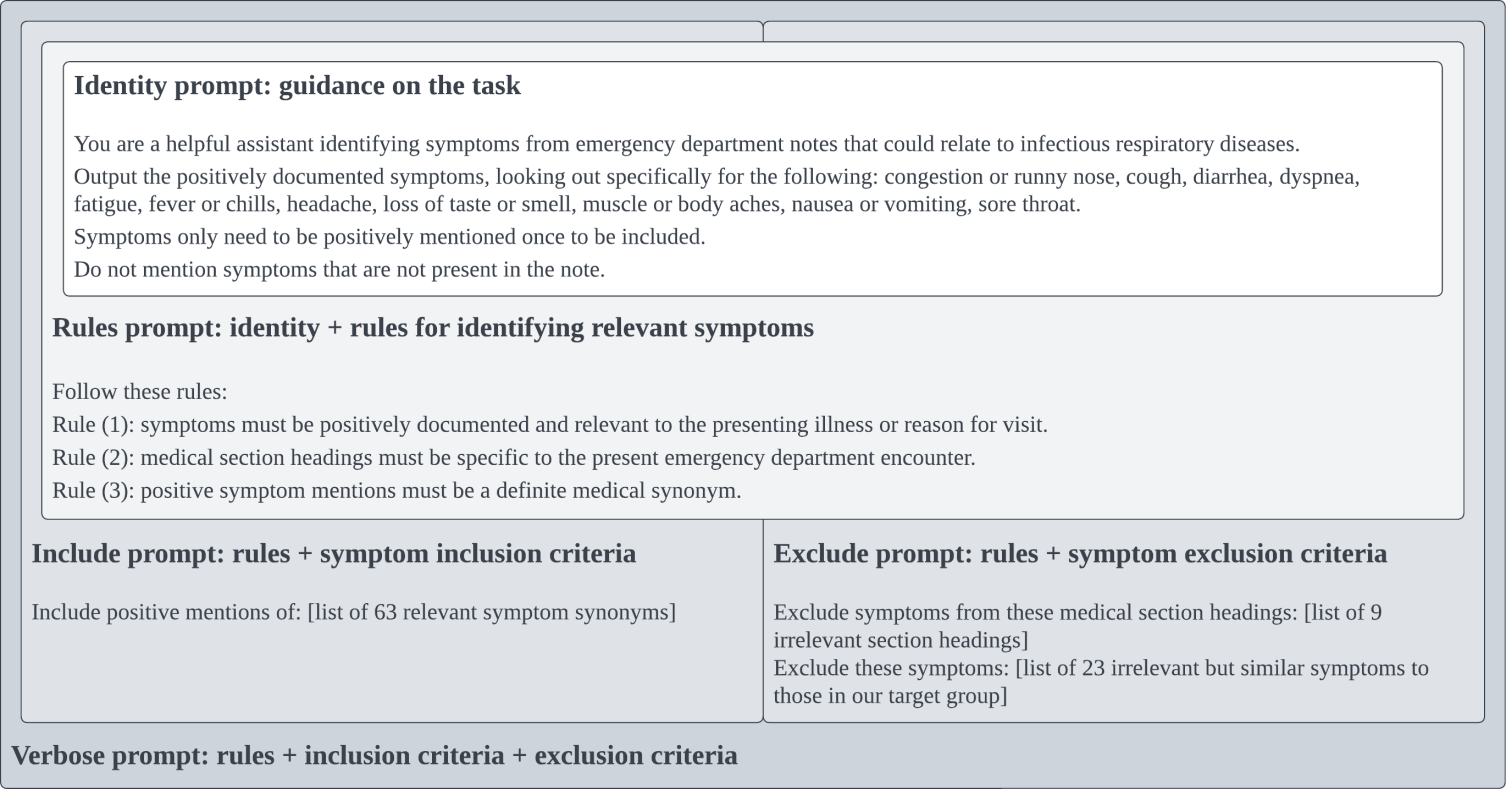
Large language model prompts intended to reproduce chart review criteria. The identity prompt contains text present in every type of prompt. The rules prompt extends the identity prompt with basic chart review criteria. Include and exclude prompts extend the rules prompt with symptom-specific criteria. The verbose prompt combines all prompts to approximate the same chart review criteria used by human subject matter experts.

For each LLM, 5 chart review prompts [45] were developed to follow symptom annotation guidelines. An overview is shown in Figure 1. Prompts ranged in complexity from an identity prompt, where LLMs were instructed to assume the identity of a chart reviewer, to a verbose prompt containing symptom-specific synonyms and inclusion and exclusion criteria. The 5 prompts were evaluated across 4 output parsing pipelines, yielding 20 prompting strategies for each LLM (Multimedia Appendix 3). All pipelines normalized LLM output into a structured CSV format containing symptoms identified in each note. Of the 4 LLM output parsing pipelines, 2 handled text and 2 handled JSON.

### Ethical Considerations

The BCH Committee on Clinical Investigation (BCH IRB-P00043392) and the Indiana University Institutional Review Board (IU IRB 24673) each determined the study to be exempt from full human participant oversight. Waivers of consent were obtained to allow corpus extraction and chart review of ED notes for institutional review board–approved study personnel. Notes were not shared between sites and not anonymized prior to LLM processing. All analyses were conducted in HIPAA-secure environments. Open-source LLMs were hosted on premises. OpenAI models were hosted by Azure under a Business Associates Agreement for HIPAA compliance. Clinical notes and patient data have been omitted from figures, tables, and appendices; only aggregate statistics are reported.

## Results

**Figure 2.**
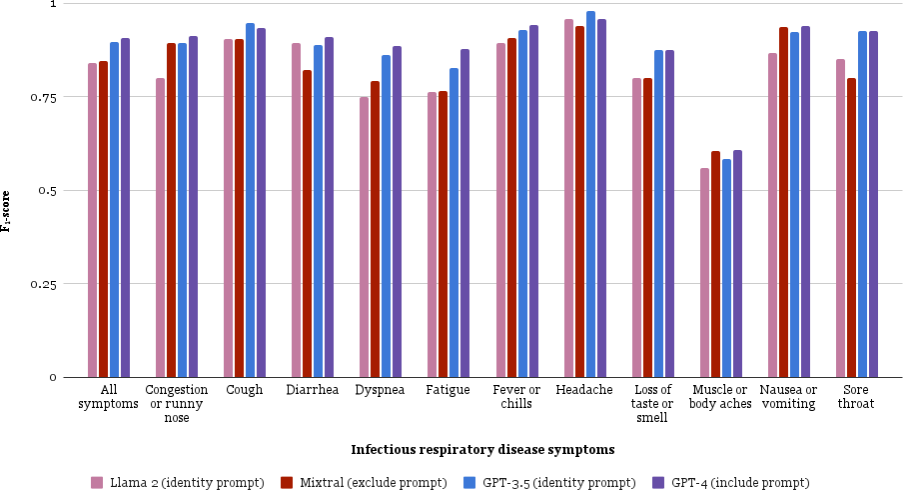
*F*_1_-scores for large language model (LLM) optimal prompt strategies using the development corpus. Each color denotes a symptom identification method using its optimal prompting strategy: Llama2 (identity prompt), Mixtral (exclude prompt), GPT-3.5 (identity prompt), and GPT-4 (include prompt). Each of the 11 infectious disease symptoms are shown as well as a summary score for all symptoms. Overall, GPT-4 performed best, with a micro *F*_1_-score of 90.8% for all symptoms combined.

Demographic characteristics of patients with notes in the study corpus are presented in Multimedia Appendix 4. Frequencies for each symptom are in Multimedia Appendix 5. Figure 2 shows symptom identification *F*_1_-scores in the development corpus using the optimal prompting strategy for each LLM. Optimal LLM instructions for chart review varied considerably among LLMs (Multimedia Appendix 6). Every LLM was optimized using the JSON output parsing pipeline.

**Figure 3.**
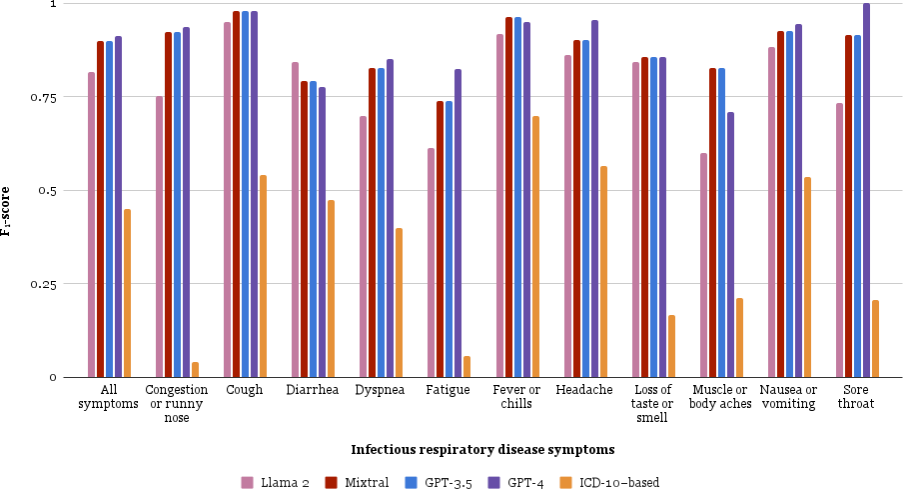
*F*_1_-scores for large language model (LLM) optimal prompt strategies using the test corpus. Each color denotes a symptom identification method: Llama2, Mixtral, GPT-3.5, GPT-4, and the *ICD-10*–based method. Each of the 11 infectious respiratory disease symptoms are shown as well as a summary score for all symptoms. GPT-4 performed best, with an overall micro *F*_1_-score of 91.4% for all symptoms combined. *ICD-10*: *International Classification of Diseases, Tenth Revision.*

The performance of each symptom identification method was evaluated with the test corpus using the *F*_1_-score statistic. The *ICD-10*–based method performed worst (*F*_1_-score=45.1%) compared to each LLM method. GPT-4 was the highest-scoring LLM (*F*_1_-score=91.4%; *P*<.001), followed by GPT-3.5 (*F*_1_-score=90.0%; *P*<.001), Llama2 (*F*_1_-score=81.7%; *P*<.001), and Mixtral (*F*_1_-score=83.5%; *P*<.001). Figure 3 shows symptom accuracy for the optimal prompting strategy of each LLM as well as the *ICD-10*–based method. Multimedia Appendix 7 contains method details and statistical results.

**Figure 4.**
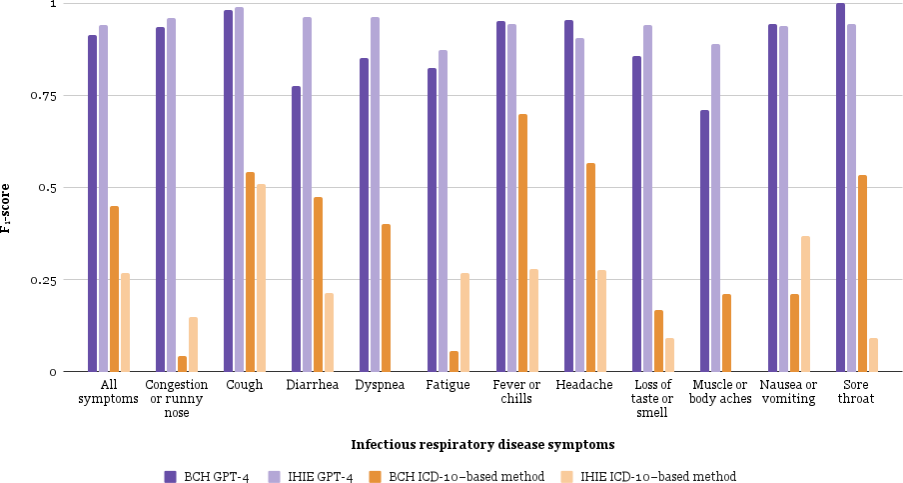
Generalizability of symptom identification accuracy across sites. One site is a large Northeastern urban pediatric academic medical center (BCH). The other is a Midwestern statewide health information exchange (IHIE) that provided data from 21 emergency departments. GPT-4 and the *ICD-10*–based method were compared. *F*_1_-scores are shown for each method and symptom benchmarked against ground truth labels from chart reviews in test and validation corpora. BCH: Boston Children’s Hospital; *ICD-10*: *International Classification of Diseases, Tenth Revision*; IHIE: Indiana Health Information Exchange.

Using the validation corpus from IHIE, GPT-4 accuracy was measured with no further model training or fine-tuning of the BCH model. Accuracy improved for GPT-4 (*F*_1_-score=94.0%; an absolute increase of 2.6%) but accuracy for the *ICD-10*–based method was worse (*F*_1_-score=26.9%; an absolute decrease of 18.2%). Generalizability from the BCH to IHIE corpus was better for GPT-4 than the *ICD-10* method (*P*<.001). Figure 4 shows that GPT-4 accuracy was higher than the *ICD-10*–based method for all symptoms at both sites. Details and results are in Multimedia Appendix 8.

## Discussion

### Principal Results

In this multisite study, LLM-based symptom identification consistently outperformed *ICD-10*–based methods for each infectious respiratory disease symptom evaluated. GPT-4 achieved the highest *F*_1_-score, and results generalized well to an external validation corpus without customization. Low accuracy for *ICD-10*–based symptom identification and variability in multisite studies are consistent with prior literature [16,18,46].

Importantly, LLM strategies all used “zero-shot” prompts and required no site-specific artificial intelligence training, fine-tuning, or ground truth examples. The potential to reduce human labor represents a major advantage of LLM methods over traditional NLP methods that require human labor to curate symptom concept dictionaries, annotate ground truth examples, and calibrate at each health care site.

### Limitations and Future Work

This study focused specifically on identifying symptoms of infectious respiratory diseases. However, generalizability of LLMs to other clinical domains and broader symptom categories remains to be validated. Furthermore, while GPT-4 performance was excellent in a validation corpus from 21 EDs, other settings, including primary care, should be studied. Other LLM models such as Google Gemini, Anthropic Claude, and DeepSeek R1 were not available for use in our HIPAA-secure settings. Future work should explore recent LLM developments. For example, the latest agentic methods could generalize to new symptom sets dynamically through multistage interactions with users.

It was beyond the scope of this study to estimate symptom prevalence in the study population. However, given outstanding LLM performance, one could approximate true prevalence from apparent prevalence in electronic health records [47]. Future work is needed to incorporate LLM-assisted chart review and pattern recognition. Doing this in real time, at a national scale, would truly improve public health efforts [3,47,48].

### Conclusions

Our findings underscore the potential of LLMs to address gaps in traditional methods to identify symptoms in health records, paving the way for advancements in syndromic biosurveillance and other use cases. LLMs can be instructed to mimic human chart reviewers with high accuracy. Future work should assess broader symptom types and health care settings.

## Supplementary material

10.2196/72984 Multimedia Appendix 1 Kappa agreement scores are shown for human expert chart reviewers at 2 sites (BCH and IHIE) At BCH, a third reviewer (AG) was available for measurement. IHIE had 2 reviewers. BCH: Boston Children’s Hospital; IHIE: Indiana Health Information Exchange.

10.2196/72984 Multimedia Appendix 2 *ICD-10* codes for symptoms of infectious respiratory disease. *ICD-10*: *International Classification of Diseases, Tenth Revision.*

10.2196/72984 Multimedia Appendix 3 Prompting templates and strategies, including verbatim prompting templates used across different large language models to conform to their instruction tuning specifications, as well as all 20 prompting strategies examined using our development corpus.

10.2196/72984 Multimedia Appendix 4 Patient demographics across sites and corpora. Demographics reported include binned age groups, administrative sex, and patient-reported race.

10.2196/72984 Multimedia Appendix 5 Frequency of suspected symptoms at the time of corpus construction. To ensure decent distribution of symptoms across each corpus, samples were based on cTAKES-annotated symptom mentions. This aimed to guarantee that, even for rare symptoms, a bare minimum of symptoms likely to be positive was included in all corpora.

10.2196/72984 Multimedia Appendix 6 Large language model (LLM) symptom identification performance using the development corpus. *F*_1_-scores are provided for all 80 combinations of models and strategies. Detailed performance results are provided for each LLM using their best performing LLM strategy.

10.2196/72984 Multimedia Appendix 7 Symptom identification performance using the test corpus from BCH and the best strategy identified for each LLM. Metrics include *F*_1_-score, sensitivity, specificity, positive predictive value, negative predictive value, as well as raw counts of true positives, false negatives, true negatives, and false positives across all symptoms individually and aggregated. McNemar significance tests compare *ICD-10*–based symptom identification to LLM-based symptom identification. BCH: Boston Children’s Hospital; *ICD-10*: *International Classification of Diseases, Tenth Revision*; LLM: large language model.

10.2196/72984 Multimedia Appendix 8 LLM symptom identification performance using the validation corpus. Sheets provided show detailed results for GPT-4 and *ICD-10* (including performance metrics and raw counts) as well as tables comparing the performance of both the validation and test corpora. McNemar significance tests compare *ICD-10*–based symptom identification to LLM-based symptom identification. *ICD-10*: *International Classification of Diseases, Tenth Revision*; LLM: large language model.

## Abbreviations

BCH: Boston Children’s Hospital
ED: emergency department
HIPAA: Health Insurance Portability and Accountability Act
*ICD-10*: *International Classification of Diseases, Tenth Revision*
IHIE: Indiana Health Information Exchange
LLM: large language model
NLP: natural language processing
