# Supplementary material for: Large Language Model Symptom Identification From Clinical Text: Multicenter Study
Source: J Med Internet Res. 2025 Jul 31;27:e72984. doi: 10.2196/72984 (PMC12313083; doi:10.2196/72984)
Supplement: Multimedia Appendix 3 [file jmir-v27-e72984-s003.pdf]

Multimedia Appendix 3. Strategies and Prompts evaluated in experiments, verbatim.

Supplementary information for: "Large Language Model Symptom Identification from Clinical Text: A Multi-Center Study"

Andrew J McMurry PhD1,2, Dylan Phelan MS1, Brian E Dixon PhD, MPA3,4, Alon Geva MD, MPH1,5, Daniel Gottlieb MPA1,6, James R Jones MPhil1, Michael Terry BS1, David Taylor BS4, Hannah Grace Callaway MS4, Sneha Manoharan MS4, Timothy Miller PhD1,2, Karen L Olson PhD1,2, Kenneth D Mandl MD MPH1,2*

1 Computational Health Informatics Program, Boston Children's Hospital, Boston, MA, USA
2 Department of Pediatrics, Harvard Medical School, Boston, MA, USA
3 Department of Health Policy and Management, Fairbanks School of Public Health, Indiana University, Indianapolis, IN, USA
4 Center for Biomedical Informatics, Regenstrief Institute, Indianapolis, IN, USA
5 Department of Anaesthesia, Harvard Medical School, Boston, MA, USA
6 Department of Biomedical Informatics, Harvard Medical School, Boston, MA, USA

*Corresponding author email: Kenneth_Mandl@harvard.edu (KDM)

Table of Contents

Multimedia Appendix 3 3
Mixtral Prompt Template 4
Llama Prompt Template 4
GPT Prompt Template 4
Rules Strategy 5
Identity Strategy 5
Include Strategy 6
Exclude Strategy 7
Verbose Strategy 8
RulesSimplification Strategy 9
IdentitySimplification Strategy 10
IncludeSimplification Strategy 11
ExcludeSimplification Strategy 12
VerboseSimplification Strategy 13
RulesJSON Strategy 15
IdentityJSON Strategy 16
IncludeJSON Strategy 17
ExcludeJSON Strategy 18
VerboseJSON Strategy 19
RulesJSONValidation Strategy 20
IdentityJSONValidation Strategy 21
IncludeJSONValidation Strategy 22
ExcludeJSONValidation Strategy 24
VerboseJSONValidation Strategy 25

Multimedia Appendix 3

Briefly, LLMs types investigated included Mixtral, Llama, and GPT (-3.5 and -4). Each LLM type was provided a Prompt Template conforming to the LLM's instruction-tuning recommendations. Each LLM was then evaluated using 20 strategies to optimize the LLM performance. The 20 strategies encompass combinations of 5 types of prompts (rules, identity, include, exclude, verbose) and 4 output post processing pipelines (basic, simplification, JSON, JSON Validation).

Three Prompt Templates were used for Mixtral, Llama, and GPT (-3.5 and -4). Prompt templates structure information for LLM in a recognized format recommended by the LLM vendor. Prompt templates were determined by reading model documentation and validated with manual testing. Templates are delimited in triple-quote blocks for readability and contain angle-bracketed VAR_X variables. These variables are dynamically populated with information based on which prompting strategy is being used to query the model.

Two pipelines generated text output from LLMs, post-processed the output using Apache cTAKES, and then normalized outputs to a CSV format. Two pipelines generated structured JSON output that was directly normalized into CSV format.

The text output "basic" pipeline instructs the LLM to summarize symptoms mentioned in notes. "Simplification with negation removal" extends the "basic" pipeline and adds an LLM step to simplify its previous output and remove symptoms that are not positively mentioned (e.g. no cough). The structured output "JSON" pipeline directly instructs the LLM to respond with JSON. "JSON validation" extends the "JSON" pipeline and attempts to correct for malformed LLM output responses with a second request to LLMs.

Below are also the twenty strategies. In strategies, angle-bracketed VAR_X variables communicate where the runtime variables would be inserted and take two forms: Clinical notes, or the output of a prior request. Each strategy consists of one or two requests to the LLM. Strategies with multiple requests are delimited with SECOND REQUEST strings for readability. The final output of a strategy is the output of the final request.

By combining prompt formats with the instructions and contexts associated with each strategy, readers can replicate the prompting used in our experiments. Additionally, all the code used in our prompting experiments is provided here: https://github.com/smart-on-fhir/infectious-symptoms-llm-study.

Mixtral Prompt Template

Note that Mixtral, at the time of writing, does not support System prompts, and thus we don't leverage them. Prompt templates were derived from documentation found at https://huggingface.co/mistralai/Mixtral-8x7B-Instruct-v0.1#instruction-format and https://www.promptingguide.ai/models/mixtral#prompt-engineering-guide-for-mixtral-8x7b

""""

[INST]<VAR_INSTRUCTION>

<VAR_CONTEXT> [/INST]

""""

Llama Prompt Template

Prompt templates were derived from documentation found at https://www.llama.com/docs/model-cards-and-prompt-formats/meta-llama-2/

And https://huggingface.co/blog/llama2#how-to-prompt-llama-2

""""

<s>[INST] <<SYS>>

<VAR_INSTRUCTION>

<</SYS>>

<VAR_CONTEXT> [/INST]

""""

GPT Prompt Template

For GPT, we leverage the AzureOpenAI client provided by the openai pypi package, found here. Leveraging their chat completions API, we provide an array of two messages: first, a simple system prompt message using the "system" role. Second is the following template populated with strategy-specific information, sent using the "user" role. Documentation for this chat completions approach can be found here: https://platform.openai.com/docs/api-reference/chat/create

"""" System Prompt Message

You are a helpful assistant

""""

"""" User Prompt Message

Instructions

<VAR_INSTRUCTION>

Text

<VAR_CONTEXT>

""""

Rules Strategy

Instruction:

You are a helpful assistant identifying symptoms from emergency department notes that could relate to infectious respiratory diseases.

Output positively documented symptoms, looking out specifically for the following: Congestion or runny nose, Cough, Diarrhea, Dyspnea, Fatigue, Fever or chills, Headache, Loss of taste or smell, Muscle or body aches, Nausea or vomiting, Sore throat.

Symptoms only need to be positively mentioned once to be included.

Do not mention symptoms that are not present in the note.

Follow these rules:

Rule (1): Symptoms must be positively documented and relevant to the presenting illness or reason for visit.

Rule (2): Medical section headings must be specific to the present emergency department encounter.

Rule (3): Positive symptom mentions must be a definite medical synonym.

Context:

<VAR_CLINICAL_NOTE>

Identity Strategy

Instruction:

You are a helpful assistant identifying symptoms from emergency department notes that could relate to infectious respiratory diseases.

Output positively documented symptoms, looking out specifically for the following: Congestion or runny nose, Cough, Diarrhea, Dyspnea, Fatigue, Fever or chills, Headache, Loss of taste or smell, Muscle or body aches, Nausea or vomiting, Sore throat.

Symptoms only need to be positively mentioned once to be included.

Do not mention symptoms that are not present in the note.

Context:

<VAR_CLINICAL_NOTE>

Include Strategy

Instruction:

You are a helpful assistant identifying symptoms from emergency department notes that could relate to infectious respiratory diseases.

Output positively documented symptoms, looking out specifically for the following: Congestion or runny nose, Cough, Diarrhea, Dyspnea, Fatigue, Fever or chills, Headache, Loss of taste or smell, Muscle or body aches, Nausea or vomiting, Sore throat.

Symptoms only need to be positively mentioned once to be included.

Do not mention symptoms that are not present in the note.

Follow these rules:

Rule (1): Symptoms must be positively documented and relevant to the presenting illness or reason for visit.

Rule (2): Medical section headings must be specific to the present emergency department encounter.

Include positive symptoms from these medical section headings: "Chief Complaint", "History of Present Illness", "HPI", "Review of Systems", "Physical Exam", "Vital Signs", "Assessment and Plan", "Medical Decision Making".

Rule (3): Positive symptom mentions must be a definite medical synonym.

Include positive mentions of: "anosmia", "loss of taste", "loss of smell", "rhinorrhea", "congestion", "discharge", "nose is dripping", "runny nose", "stuffy nose", "cough", "tussive or post-tussive", "cough is unproductive", "productive cough", "dry cough", "wet cough", "producing sputum", "diarrhea", "watery stool", "fatigue", "tired", "exhausted", "weary", "malaise", "feeling generally unwell", "fever", "pyrexia", "chills", "temperature greater than or equal 100.4 Fahrenheit or 38 celsius", "Temperature >= 100.4F", "Temperature >= 38C", "headache", "HA", "migraine", "cephalgia", "head pain", "muscle or body aches", "muscle aches", "generalized aches and pains", "body aches", "myalgias", "myoneuralgia", "soreness", "generalized aches and pains", "nausea or vomiting", "Nausea", "vomiting", "emesis", "throwing up", "queasy", "regurgitated", "shortness of breath", "difficulty breathing", "SOB", "Dyspnea", "breathing is short", "increased breathing", "labored breathing", "distressed breathing", "sore throat", "throat pain", "pharyngeal pain", "pharyngitis", "odynophagia".

Context:

<VAR_CLINICAL_NOTE>

Exclude Strategy

Instruction:

You are a helpful assistant identifying symptoms from emergency department notes that could relate to infectious respiratory diseases.

Output positively documented symptoms, looking out specifically for the following: Congestion or runny nose, Cough, Diarrhea, Dyspnea, Fatigue, Fever or chills, Headache, Loss of taste or smell, Muscle or body aches, Nausea or vomiting, Sore throat.

Symptoms only need to be positively mentioned once to be included.

Do not mention symptoms that are not present in the note.

Follow these rules:

Rule (1): Symptoms must be positively documented and relevant to the presenting illness or reason for visit.

Rule (2): Medical section headings must be specific to the present emergency department encounter.

Exclude symptoms from these medical section headings: "Past Medical History", "PMHFamily History", "FHX", "Social History", "SHX", "Medications", "Allergies", "Imaging", "Diagnostic Study".Rule (3): Positive symptom mentions must be a definite medical synonym.

Exclude these symptoms: "injury related to loss of taste or smell", "wheezing", "crackles", "croup", "loose stool", "bloody stool", "looked ill", "afebrile", "felt warm", "headache due to injury", "localized pain", "injury", "abdominal pain", "lower back pain", "gastritis", "gastroparesis", "BiPAP", "CPAP", "oxygen need", "streptococcus", "dysphagia", "hoarseness", "red throat".

Context:

<VAR_CLINICAL_NOTE>

Verbose Strategy

Instruction:

You are a helpful assistant identifying symptoms from emergency department notes that could relate to infectious respiratory diseases.

Output positively documented symptoms, looking out specifically for the following: Congestion or runny nose, Cough, Diarrhea, Dyspnea, Fatigue, Fever or chills, Headache, Loss of taste or smell, Muscle or body aches, Nausea or vomiting, Sore throat.

Symptoms only need to be positively mentioned once to be included.

Do not mention symptoms that are not present in the note.

Follow these rules:

Rule (1): Symptoms must be positively documented and relevant to the presenting illness or reason for visit.

Rule (2): Medical section headings must be specific to the present emergency department encounter.

Include positive symptoms from these medical section headings: "Chief Complaint", "History of Present Illness", "HPI", "Review of Systems", "Physical Exam", "Vital Signs", "Assessment and Plan", "Medical Decision Making".

Exclude all symptoms from these medical section headings: "Past Medical History", "PMHFamily History", "FHX", "Social History", "SHX", "Medications", "Allergies", "Imaging", "Diagnostic Study".

Rule (3): Positive symptom mentions must be a definite medical synonym.

Include positive mentions of these medical terms: "anosmia", "loss of taste", "loss of smell", "rhinorrhea", "congestion", "discharge", "nose is dripping", "runny nose", "stuffy nose", "cough", "tussive or post-tussive", "cough is unproductive", "productive cough", "dry cough", "wet cough", "producing sputum", "diarrhea", "watery stool", "fatigue", "tired", "exhausted", "weary", "malaise", "feeling generally unwell", "fever", "pyrexia", "chills", "temperature greater than or equal 100.4 Fahrenheit or 38 celsius", "Temperature >= 100.4F", "Temperature >= 38C", "headache", "HA", "migraine", "cephalgia", "head pain", "muscle or body aches", "muscle aches", "generalized aches and pains", "body aches", "myalgias", "myoneuralgia", "soreness", "generalized aches and pains", "nausea or vomiting", "Nausea", "vomiting", "emesis", "throwing up", "queasy", "regurgitated", "shortness of breath", "difficulty breathing", "SOB", "Dyspnea", "breathing is short", "increased breathing", "labored breathing", "distressed breathing", "sore throat", "throat pain", "pharyngeal pain", "pharyngitis", "odynophagia".

Exclude these symptoms: "injury related to loss of taste or smell", "wheezing", "crackles", "croup", "loose stool", "bloody stool", "looked ill", "afebrile", "felt warm", "headache due to injury", "localized pain", "injury", "abdominal pain", "lower back pain", "gastritis", "gastroparesis", "BiPAP", "CPAP", "oxygen need", "streptococcus", "dysphagia", "hoarseness", "red throat".

Context:

<VAR_CLINICAL_NOTE>

RulesSimplification Strategy

Instruction:

You are a helpful assistant identifying symptoms from emergency department notes that could relate to infectious respiratory diseases.

Output positively documented symptoms, looking out specifically for the following: Congestion or runny nose, Cough, Diarrhea, Dyspnea, Fatigue, Fever or chills, Headache, Loss of taste or smell, Muscle or body aches, Nausea or vomiting, Sore throat.

Symptoms only need to be positively mentioned once to be included.

Do not mention symptoms that are not present in the note.

Follow these rules:

Rule (1): Symptoms must be positively documented and relevant to the presenting illness or reason for visit.

Rule (2): Medical section headings must be specific to the present emergency department encounter.

Rule (3): Positive symptom mentions must be a definite medical synonym.

Context:

<VAR_CLINICAL_NOTE>

===SECOND REQUEST===

Instruction:

You are an expert editor reviewing a clinical note summary.

The previous reviewer may have included irrelevant, negative symptoms in their summarization.

Simplify repetitive information found in this summary, and remove mentions of negative symptoms from this summary (e.g. "No X, No recent Y, No recent changes in Z").

ONLY reply with your new summary. Do NOT explain your answers.

Context:

<VAR_PREVIOUS_SUMMARY>

IdentitySimplification Strategy

Instruction:

> You are a helpful assistant identifying symptoms from emergency department notes that could relate to infectious respiratory diseases.
>
> Output positively documented symptoms, looking out specifically for the following: Congestion or runny nose, Cough, Diarrhea, Dyspnea, Fatigue, Fever or chills, Headache, Loss of taste or smell, Muscle or body aches, Nausea or vomiting, Sore throat.
>
> Symptoms only need to be positively mentioned once to be included.
>
> Do not mention symptoms that are not present in the note.

Context:

> <VAR_CLINICAL_NOTE>

===SECOND REQUEST===

Instruction:

> You are an expert editor reviewing a clinical note summary.
>
> The previous reviewer may have included irrelevant, negative symptoms in their summarization.
>
> Simplify repetitive information found in this summary, and remove mentions of negative symptoms from this summary (e.g. "No X, No recent Y, No recent changes in Z").
>
> ONLY reply with your new summary. Do NOT explain your answers.

Context:

> <VAR_PREVIOUS_SUMMARY>

IncludeSimplification Strategy

Instruction:

You are a helpful assistant identifying symptoms from emergency department notes that could relate to infectious respiratory diseases.

Output positively documented symptoms, looking out specifically for the following: Congestion or runny nose, Cough, Diarrhea, Dyspnea, Fatigue, Fever or chills, Headache, Loss of taste or smell, Muscle or body aches, Nausea or vomiting, Sore throat.

Symptoms only need to be positively mentioned once to be included.

Do not mention symptoms that are not present in the note.

Follow these rules:

Rule (1): Symptoms must be positively documented and relevant to the presenting illness or reason for visit.

Rule (2): Medical section headings must be specific to the present emergency department encounter.

Include positive symptoms from these medical section headings: "Chief Complaint", "History of Present Illness", "HPI", "Review of Systems", "Physical Exam", "Vital Signs", "Assessment and Plan", "Medical Decision Making".

Rule (3): Positive symptom mentions must be a definite medical synonym.

Include positive mentions of: "anosmia", "loss of taste", "loss of smell", "rhinorrhea", "congestion", "discharge", "nose is dripping", "runny nose", "stuffy nose", "cough", "tussive or post-tussive", "cough is unproductive", "productive cough", "dry cough", "wet cough", "producing sputum", "diarrhea", "watery stool", "fatigue", "tired", "exhausted", "weary", "malaise", "feeling generally unwell", "fever", "pyrexia", "chills", "temperature greater than or equal 100.4 Fahrenheit or 38 celsius", "Temperature >= 100.4F", "Temperature >= 38C", "headache", "HA", "migraine", "cephalgia", "head pain", "muscle or body aches", "muscle aches", "generalized aches and pains", "body aches", "myalgias", "myoneuralgia", "soreness", "generalized aches and pains", "nausea or vomiting", "Nausea", "vomiting", "emesis", "throwing up", "queasy", "regurgitated", "shortness of breath", "difficulty breathing", "SOB", "Dyspnea", "breathing is short", "increased breathing", "labored breathing", "distressed breathing", "sore throat", "throat pain", "pharyngeal pain", "pharyngitis", "odynophagia".

Context:

<VAR_CLINICAL_NOTE>

===SECOND REQUEST===

Instruction:

You are an expert editor reviewing a clinical note summary.

The previous reviewer may have included irrelevant, negative symptoms in their summarization.

Simplify repetitive information found in this summary, and remove mentions of negative symptoms from this summary (e.g. "No X, No recent Y, No recent changes in Z").

ONLY reply with your new summary. Do NOT explain your answers.

Context:

<VAR_PREVIOUS_SUMMARY>

ExcludeSimplification Strategy

Instruction:

You are a helpful assistant identifying symptoms from emergency department notes that could relate to infectious respiratory diseases.

Output positively documented symptoms, looking out specifically for the following: Congestion or runny nose, Cough, Diarrhea, Dyspnea, Fatigue, Fever or chills, Headache, Loss of taste or smell, Muscle or body aches, Nausea or vomiting, Sore throat.

Symptoms only need to be positively mentioned once to be included.

Do not mention symptoms that are not present in the note.

Follow these rules:

Rule (1): Symptoms must be positively documented and relevant to the presenting illness or reason for visit.

Rule (2): Medical section headings must be specific to the present emergency department encounter.

Exclude symptoms from these medical section headings: "Past Medical History", "PMHFamily History", "FHX", "Social History", "SHX", "Medications", "Allergies", "Imaging", "Diagnostic Study".Rule (3): Positive symptom mentions must be a definite medical synonym.

Exclude these symptoms: "injury related to loss of taste or smell", "wheezing", "crackles", "croup", "loose stool", "bloody stool", "looked ill", "afebrile", "felt warm", "headache due to injury", "localized pain", "injury", "abdominal pain", "lower back pain", "gastritis", "gastroparesis", "BiPAP", "CPAP", "oxygen need", "streptococcus", "dysphagia", "hoarseness", "red throat".

Context:

<VAR_CLINICAL_NOTE>

===SECOND REQUEST===

Instruction:

You are an expert editor reviewing a clinical note summary.

The previous reviewer may have included irrelevant, negative symptoms in their summarization.

Simplify repetitive information found in this summary, and remove mentions of negative symptoms from this summary (e.g. "No X, No recent Y, No recent changes in Z").

ONLY reply with your new summary. Do NOT explain your answers.

Context:

<VAR_PREVIOUS_SUMMARY>

VerboseSimplification Strategy

Instruction:

You are a helpful assistant identifying symptoms from emergency department notes that could relate to infectious respiratory diseases.

Output positively documented symptoms, looking out specifically for the following: Congestion or runny nose, Cough, Diarrhea, Dyspnea, Fatigue, Fever or chills, Headache, Loss of taste or smell, Muscle or body aches, Nausea or vomiting, Sore throat.

Symptoms only need to be positively mentioned once to be included.

Do not mention symptoms that are not present in the note.

Follow these rules:

Rule (1): Symptoms must be positively documented and relevant to the presenting illness or reason for visit.

Rule (2): Medical section headings must be specific to the present emergency department encounter.

Include positive symptoms from these medical section headings: "Chief Complaint", "History of Present Illness", "HPI", "Review of Systems", "Physical Exam", "Vital Signs", "Assessment and Plan", "Medical Decision Making".

Exclude all symptoms from these medical section headings: "Past Medical History", "PMHFamily History", "FHX", "Social History", "SHX", "Medications", "Allergies", "Imaging", "Diagnostic Study".

Rule (3): Positive symptom mentions must be a definite medical synonym.

Include positive mentions of these medical terms: "anosmia", "loss of taste", "loss of smell", "rhinorrhea", "congestion", "discharge", "nose is dripping", "runny nose", "stuffy nose", "cough", "tussive or post-tussive", "cough is unproductive", "productive cough", "dry cough", "wet cough", "producing sputum", "diarrhea", "watery stool", "fatigue", "tired", "exhausted", "weary", "malaise", "feeling generally unwell", "fever", "pyrexia", "chills", "temperature greater than or equal 100.4 Fahrenheit or 38 celsius", "Temperature >= 100.4F", "Temperature >= 38C", "headache", "HA", "migraine", "cephalgia", "head pain", "muscle or body aches", "muscle aches", "generalized aches and pains", "body aches", "myalgias", "myoneuralgia", "soreness", "generalized aches and pains", "nausea or vomiting", "Nausea", "vomiting", "emesis", "throwing up", "queasy", "regurgitated", "shortness of breath", "difficulty breathing", "SOB", "Dyspnea", "breathing is short", "increased breathing", "labored breathing", "distressed breathing", "sore throat", "throat pain", "pharyngeal pain", "pharyngitis", "odynophagia".

Exclude these symptoms: "injury related to loss of taste or smell", "wheezing", "crackles", "croup", "loose stool", "bloody stool", "looked ill", "afebrile", "felt warm", "headache due to injury", "localized pain", "injury", "abdominal pain", "lower back pain", "gastritis", "gastroparesis", "BiPAP", "CPAP", "oxygen need", "streptococcus", "dysphagia", "hoarseness", "red throat".

Context:

<VAR_CLINICAL_NOTE>

===SECOND REQUEST===

Instruction:

You are an expert editor reviewing a clinical note summary.

The previous reviewer may have included irrelevant, negative symptoms in their summarization.

Simplify repetitive information found in this summary, and remove mentions of negative symptoms from this summary (e.g. "No X, No recent Y, No recent changes in Z").

ONLY reply with your new summary. Do NOT explain your answers.

Context:

<VAR_PREVIOUS_SUMMARY>

RulesJSON Strategy

Instruction:

You are a helpful assistant identifying symptoms from emergency department notes that could relate to infectious respiratory diseases.

Output positively documented symptoms, looking out specifically for the following: Congestion or runny nose, Cough, Diarrhea, Dyspnea, Fatigue, Fever or chills, Headache, Loss of taste or smell, Muscle or body aches, Nausea or vomiting, Sore throat.

Symptoms only need to be positively mentioned once to be included.

Do not mention symptoms that are not present in the note.

Follow these rules:

Rule (1): Symptoms must be positively documented and relevant to the presenting illness or reason for visit.

Rule (2): Medical section headings must be specific to the present emergency department encounter.

Rule (3): Positive symptom mentions must be a definite medical synonym.

Your reply must be parsable as JSON.

Format your response using only the following JSON schema: {"Congestion or runny nose": boolean, "Cough": boolean, "Diarrhea": boolean, "Dyspnea": boolean, "Fatigue": boolean, "Fever or chills": boolean, "Headache": boolean, "Loss of taste or smell": boolean, "Muscle or body aches": boolean, "Nausea or vomiting": boolean, "Sore throat": boolean}. Each JSON key should correspond to a symptom, and each value should be true if that symptom is indicated in the clinical note; false otherwise.

Never explain yourself, and only reply with JSON.

Context:

<VAR_CLINICAL_NOTE>

IdentityJSON Strategy

Instruction:

You are a helpful assistant identifying symptoms from emergency department notes that could relate to infectious respiratory diseases.

Output positively documented symptoms, looking out specifically for the following: Congestion or runny nose, Cough, Diarrhea, Dyspnea, Fatigue, Fever or chills, Headache, Loss of taste or smell, Muscle or body aches, Nausea or vomiting, Sore throat.

Symptoms only need to be positively mentioned once to be included.

Do not mention symptoms that are not present in the note.

Your reply must be parsable as JSON.

Format your response using only the following JSON schema: {"Congestion or runny nose": boolean, "Cough": boolean, "Diarrhea": boolean, "Dyspnea": boolean, "Fatigue": boolean, "Fever or chills": boolean, "Headache": boolean, "Loss of taste or smell": boolean, "Muscle or body aches": boolean, "Nausea or vomiting": boolean, "Sore throat": boolean}. Each JSON key should correspond to a symptom, and each value should be true if that symptom is indicated in the clinical note; false otherwise.

Never explain yourself, and only reply with JSON.

Context:

<VAR_CLINICAL_NOTE>

IncludeJSON Strategy

Instruction:

You are a helpful assistant identifying symptoms from emergency department notes that could relate to infectious respiratory diseases.

Output positively documented symptoms, looking out specifically for the following: Congestion or runny nose, Cough, Diarrhea, Dyspnea, Fatigue, Fever or chills, Headache, Loss of taste or smell, Muscle or body aches, Nausea or vomiting, Sore throat.

Symptoms only need to be positively mentioned once to be included.

Do not mention symptoms that are not present in the note.

Follow these rules:

Rule (1): Symptoms must be positively documented and relevant to the presenting illness or reason for visit.

Rule (2): Medical section headings must be specific to the present emergency department encounter.

Include positive symptoms from these medical section headings: "Chief Complaint", "History of Present Illness", "HPI", "Review of Systems", "Physical Exam", "Vital Signs", "Assessment and Plan", "Medical Decision Making".

Rule (3): Positive symptom mentions must be a definite medical synonym.

Include positive mentions of: "anosmia", "loss of taste", "loss of smell", "rhinorrhea", "congestion", "discharge", "nose is dripping", "runny nose", "stuffy nose", "cough", "tussive or post-tussive", "cough is unproductive", "productive cough", "dry cough", "wet cough", "producing sputum", "diarrhea", "watery stool", "fatigue", "tired", "exhausted", "weary", "malaise", "feeling generally unwell", "fever", "pyrexia", "chills", "temperature greater than or equal 100.4 Fahrenheit or 38 celsius", "Temperature >= 100.4F", "Temperature >= 38C", "headache", "HA", "migraine", "cephalgia", "head pain", "muscle or body aches", "muscle aches", "generalized aches and pains", "body aches", "myalgias", "myoneuralgia", "soreness", "generalized aches and pains", "nausea or vomiting", "Nausea", "vomiting", "emesis", "throwing up", "queasy", "regurgitated", "shortness of breath", "difficulty breathing", "SOB", "Dyspnea", "breathing is short", "increased breathing", "labored breathing", "distressed breathing", "sore throat", "throat pain", "pharyngeal pain", "pharyngitis", "odynophagia".

Your reply must be parsable as JSON.

Format your response using only the following JSON schema: {"Congestion or runny nose": boolean, "Cough": boolean, "Diarrhea": boolean, "Dyspnea": boolean, "Fatigue": boolean, "Fever or chills": boolean, "Headache": boolean, "Loss of taste or smell": boolean, "Muscle or body aches": boolean, "Nausea or vomiting": boolean, "Sore throat": boolean}. Each JSON key should correspond to a symptom, and each value should be true if that symptom is indicated in the clinical note; false otherwise.

Never explain yourself, and only reply with JSON.

Context:

<VAR_CLINICAL_NOTE>

ExcludeJSON Strategy

Instruction:

You are a helpful assistant identifying symptoms from emergency department notes that could relate to infectious respiratory diseases.

Output positively documented symptoms, looking out specifically for the following: Congestion or runny nose, Cough, Diarrhea, Dyspnea, Fatigue, Fever or chills, Headache, Loss of taste or smell, Muscle or body aches, Nausea or vomiting, Sore throat.

Symptoms only need to be positively mentioned once to be included.

Do not mention symptoms that are not present in the note.

Follow these rules:

Rule (1): Symptoms must be positively documented and relevant to the presenting illness or reason for visit.

Rule (2): Medical section headings must be specific to the present emergency department encounter.

Exclude symptoms from these medical section headings: "Past Medical History", "PMHFamily History", "FHX", "Social History", "SHX", "Medications", "Allergies", "Imaging", "Diagnostic Study".Rule (3): Positive symptom mentions must be a definite medical synonym.

Exclude these symptoms: "injury related to loss of taste or smell", "wheezing", "crackles", "croup", "loose stool", "bloody stool", "looked ill", "afebrile", "felt warm", "headache due to injury", "localized pain", "injury", "abdominal pain", "lower back pain", "gastritis", "gastroparesis", "BiPAP", "CPAP", "oxygen need", "streptococcus", "dysphagia", "hoarseness", "red throat".

Your reply must be parsable as JSON.

Format your response using only the following JSON schema: {"Congestion or runny nose": boolean, "Cough": boolean, "Diarrhea": boolean, "Dyspnea": boolean, "Fatigue": boolean, "Fever or chills": boolean, "Headache": boolean, "Loss of taste or smell": boolean, "Muscle or body aches": boolean, "Nausea or vomiting": boolean, "Sore throat": boolean}. Each JSON key should correspond to a symptom, and each value should be true if that symptom is indicated in the clinical note; false otherwise.

Never explain yourself and only reply with JSON.

Context:

<VAR_CLINICAL_NOTE>

VerboseJSON Strategy

Instruction:

You are a helpful assistant identifying symptoms from emergency department notes that could relate to infectious respiratory diseases.

Output positively documented symptoms, looking out specifically for the following: Congestion or runny nose, Cough, Diarrhea, Dyspnea, Fatigue, Fever or chills, Headache, Loss of taste or smell, Muscle or body aches, Nausea or vomiting, Sore throat.

Symptoms only need to be positively mentioned once to be included.

Do not mention symptoms that are not present in the note.

Follow these rules:

Rule (1): Symptoms must be positively documented and relevant to the presenting illness or reason for visit.

Rule (2): Medical section headings must be specific to the present emergency department encounter.

Include positive symptoms from these medical section headings: "Chief Complaint", "History of Present Illness", "HPI", "Review of Systems", "Physical Exam", "Vital Signs", "Assessment and Plan", "Medical Decision Making".

Exclude all symptoms from these medical section headings: "Past Medical History", "PMHFamily History", "FHX", "Social History", "SHX", "Medications", "Allergies", "Imaging", "Diagnostic Study".

Rule (3): Positive symptom mentions must be a definite medical synonym.

Include positive mentions of these medical terms: "anosmia", "loss of taste", "loss of smell", "rhinorrhea", "congestion", "discharge", "nose is dripping", "runny nose", "stuffy nose", "cough", "tussive or post-tussive", "cough is unproductive", "productive cough", "dry cough", "wet cough", "producing sputum", "diarrhea", "watery stool", "fatigue", "tired", "exhausted", "weary", "malaise", "feeling generally unwell", "fever", "pyrexia", "chills", "temperature greater than or equal 100.4 Fahrenheit or 38 celsius", "Temperature >= 100.4F", "Temperature >= 38C", "headache", "HA", "migraine", "cephalgia", "head pain", "muscle or body aches", "muscle aches", "generalized aches and pains", "body aches", "myalgias", "myoneuralgia", "soreness", "generalized aches and pains", "nausea or vomiting", "Nausea", "vomiting", "emesis", "throwing up", "queasy", "regurgitated", "shortness of breath", "difficulty breathing", "SOB", "Dyspnea", "breathing is short", "increased breathing", "labored breathing", "distressed breathing", "sore throat", "throat pain", "pharyngeal pain", "pharyngitis", "odynophagia".

Exclude these symptoms: "injury related to loss of taste or smell", "wheezing", "crackles", "croup", "loose stool", "bloody stool", "looked ill", "afebrile", "felt warm", "headache due to injury", "localized pain", "injury", "abdominal pain", "lower back pain", "gastritis", "gastroparesis", "BiPAP", "CPAP", "oxygen need", "streptococcus", "dysphagia", "hoarseness", "red throat".

Your reply must be parsable as JSON.

Format your response using only the following JSON schema: {"Congestion or runny nose": boolean, "Cough": boolean, "Diarrhea": boolean, "Dyspnea": boolean, "Fatigue": boolean, "Fever or chills": boolean, "Headache": boolean, "Loss of taste or smell": boolean, "Muscle or body aches": boolean, "Nausea or vomiting": boolean, "Sore throat": boolean}. Each JSON key should correspond to a symptom, and each value should be true if that symptom is indicated in the clinical note; false otherwise.

Never explain yourself, and only reply with JSON.

Context:

<VAR_CLINICAL_NOTE>

RulesJSONValidation Strategy

Instruction:

You are a helpful assistant identifying symptoms from emergency department notes that could relate to infectious respiratory diseases.

Output positively documented symptoms, looking out specifically for the following: Congestion or runny nose, Cough, Diarrhea, Dyspnea, Fatigue, Fever or chills, Headache, Loss of taste or smell, Muscle or body aches, Nausea or vomiting, Sore throat.

Symptoms only need to be positively mentioned once to be included.

Do not mention symptoms that are not present in the note.

Follow these rules:

Rule (1): Symptoms must be positively documented and relevant to the presenting illness or reason for visit.

Rule (2): Medical section headings must be specific to the present emergency department encounter.

Rule (3): Positive symptom mentions must be a definite medical synonym.

Your reply must be parsable as JSON.

Format your response using only the following JSON schema: {"Congestion or runny nose": boolean, "Cough": boolean, "Diarrhea": boolean, "Dyspnea": boolean, "Fatigue": boolean, "Fever or chills": boolean, "Headache": boolean, "Loss of taste or smell": boolean, "Muscle or body aches": boolean, "Nausea or vomiting": boolean, "Sore throat": boolean}. Each JSON key should correspond to a symptom, and each value should be true if that symptom is indicated in the clinical note; false otherwise.

Never explain yourself, and only reply with JSON.

Context:

<VAR_CLINICAL_NOTE>

===SECOND REQUEST===

Instruction:

If the following text is parseable, valid JSON, do nothing and return the text as is.

Otherwise, remove all non-JSON information. Then format the text so that it's valid, parsable JSON that conforms to the following JSON schema: {"Congestion or runny nose": boolean, "Cough": boolean, "Diarrhea": boolean, "Dyspnea": boolean, "Fatigue": boolean, "Fever or chills": boolean, "Headache": boolean, "Loss of taste or smell": boolean, "Muscle or body aches": boolean, "Nausea or vomiting": boolean, "Sore throat": boolean}

Never explain yourself, and only reply with JSON.

Context:

<VAR_PREVIOUS_JSON_OUTPUT>

IdentityJSONValidation Strategy

Instruction:

You are a helpful assistant identifying symptoms from emergency department notes that could relate to infectious respiratory diseases.

Output positively documented symptoms, looking out specifically for the following: Congestion or runny nose, Cough, Diarrhea, Dyspnea, Fatigue, Fever or chills, Headache, Loss of taste or smell, Muscle or body aches, Nausea or vomiting, Sore throat.

Symptoms only need to be positively mentioned once to be included.

Do not mention symptoms that are not present in the note.

Your reply must be parsable as JSON.

Format your response using only the following JSON schema: {"Congestion or runny nose": boolean, "Cough": boolean, "Diarrhea": boolean, "Dyspnea": boolean, "Fatigue": boolean, "Fever or chills": boolean, "Headache": boolean, "Loss of taste or smell": boolean, "Muscle or body aches": boolean, "Nausea or vomiting": boolean, "Sore throat": boolean}. Each JSON key should correspond to a symptom, and each value should be true if that symptom is indicated in the clinical note; false otherwise.

Never explain yourself, and only reply with JSON.

Context:

<VAR_CLINICAL_NOTE>

===SECOND REQUEST===

Instruction:

If the following text is parseable, valid JSON, do nothing and return the text as is.

Otherwise, remove all non-JSON information. Then format the text so that it's valid, parsable JSON that conforms to the following JSON schema: {"Congestion or runny nose": boolean, "Cough": boolean, "Diarrhea": boolean, "Dyspnea": boolean, "Fatigue": boolean, "Fever or chills": boolean, "Headache": boolean, "Loss of taste or smell": boolean, "Muscle or body aches": boolean, "Nausea or vomiting": boolean, "Sore throat": boolean}

Never explain yourself, and only reply with JSON.

Context:

<VAR_PREVIOUS_JSON_OUTPUT>

IncludeJSONValidation Strategy

Instruction:

You are a helpful assistant identifying symptoms from emergency department notes that could relate to infectious respiratory diseases.

Output positively documented symptoms, looking out specifically for the following: Congestion or runny nose, Cough, Diarrhea, Dyspnea, Fatigue, Fever or chills, Headache, Loss of taste or smell, Muscle or body aches, Nausea or vomiting, Sore throat.

Symptoms only need to be positively mentioned once to be included.

Do not mention symptoms that are not present in the note.

Follow these rules:

Rule (1): Symptoms must be positively documented and relevant to the presenting illness or reason for visit.

Rule (2): Medical section headings must be specific to the present emergency department encounter.

Include positive symptoms from these medical section headings: "Chief Complaint", "History of Present Illness", "HPI", "Review of Systems", "Physical Exam", "Vital Signs", "Assessment and Plan", "Medical Decision Making".

Rule (3): Positive symptom mentions must be a definite medical synonym.

Include positive mentions of: "anosmia", "loss of taste", "loss of smell", "rhinorrhea", "congestion", "discharge", "nose is dripping", "runny nose", "stuffy nose", "cough", "tussive or post-tussive", "cough is unproductive", "productive cough", "dry cough", "wet cough", "producing sputum", "diarrhea", "watery stool", "fatigue", "tired", "exhausted", "weary", "malaise", "feeling generally unwell", "fever", "pyrexia", "chills", "temperature greater than or equal 100.4 Fahrenheit or 38 celsius", "Temperature >= 100.4F", "Temperature >= 38C", "headache", "HA", "migraine", "cephalgia", "head pain", "muscle or body aches", "muscle aches", "generalized aches and pains", "body aches", "myalgias", "myoneuralgia", "soreness", "generalized aches and pains", "nausea or vomiting", "Nausea", "vomiting", "emesis", "throwing up", "queasy", "regurgitated", "shortness of breath", "difficulty breathing", "SOB", "Dyspnea", "breathing is short", "increased breathing", "labored breathing", "distressed breathing", "sore throat", "throat pain", "pharyngeal pain", "pharyngitis", "odynophagia".

Your reply must be parsable as JSON.

Format your response using only the following JSON schema: {"Congestion or runny nose": boolean, "Cough": boolean, "Diarrhea": boolean, "Dyspnea": boolean, "Fatigue": boolean, "Fever or chills": boolean, "Headache": boolean, "Loss of taste or smell": boolean, "Muscle or body aches": boolean, "Nausea or vomiting": boolean, "Sore throat": boolean}. Each JSON key should correspond to a symptom, and each value should be true if that symptom is indicated in the clinical note; false otherwise.

Never explain yourself, and only reply with JSON.

Context:

<VAR_CLINICAL_NOTE>

===SECOND REQUEST===

Instruction:

If the following text is parseable, valid JSON, do nothing and return the text as is.

Otherwise, remove all non-JSON information. Then format the text so that it's valid, parsable JSON that conforms to the following JSON schema: {"Congestion or runny nose": boolean, "Cough": boolean, "Diarrhea": boolean, "Dyspnea": boolean, "Fatigue": boolean, "Fever or chills": boolean, "Headache": boolean, "Loss of taste or smell": boolean, "Muscle or body aches": boolean, "Nausea or vomiting": boolean, "Sore throat": boolean}

Never explain yourself, and only reply with JSON.

Context:

<VAR_PREVIOUS_JSON_OUTPUT>

ExcludeJSONValidation Strategy

Instruction:

> You are a helpful assistant identifying symptoms from emergency department notes that could relate to infectious respiratory diseases.
>
> Output positively documented symptoms, looking out specifically for the following: Congestion or runny nose, Cough, Diarrhea, Dyspnea, Fatigue, Fever or chills, Headache, Loss of taste or smell, Muscle or body aches, Nausea or vomiting, Sore throat.
>
> Symptoms only need to be positively mentioned once to be included.
>
> Do not mention symptoms that are not present in the note.
>
> Follow these rules:
>
> Rule (1): Symptoms must be positively documented and relevant to the presenting illness or reason for visit.
>
> Rule (2): Medical section headings must be specific to the present emergency department encounter.
>
> Exclude symptoms from these medical section headings: "Past Medical History", "PMHFamily History", "FHX", "Social History", "SHX", "Medications", "Allergies", "Imaging", "Diagnostic Study".Rule (3): Positive symptom mentions must be a definite medical synonym.
>
> Exclude these symptoms: "injury related to loss of taste or smell", "wheezing", "crackles", "croup", "loose stool", "bloody stool", "looked ill", "afebrile", "felt warm", "headache due to injury", "localized pain", "injury", "abdominal pain", "lower back pain", "gastritis", "gastroparesis", "BiPAP", "CPAP", "oxygen need", "streptococcus", "dysphagia", "hoarseness", "red throat".
>
> Your reply must be parsable as JSON.
>
> Format your response using only the following JSON schema: {"Congestion or runny nose": boolean, "Cough": boolean, "Diarrhea": boolean, "Dyspnea": boolean, "Fatigue": boolean, "Fever or chills": boolean, "Headache": boolean, "Loss of taste or smell": boolean, "Muscle or body aches": boolean, "Nausea or vomiting": boolean, "Sore throat": boolean}. Each JSON key should correspond to a symptom, and each value should be true if that symptom is indicated in the clinical note; false otherwise.
>
> Never explain yourself, and only reply with JSON.

Context:

> <VAR_CLINICAL_NOTE>

===SECOND REQUEST===

Instruction:

> If the following text is parseable, valid JSON, do nothing and return the text as is.
>
> Otherwise, remove all non-JSON information. Then format the text so that it's valid, parsable JSON that conforms to the following JSON schema: {"Congestion or runny nose": boolean, "Cough": boolean, "Diarrhea": boolean, "Dyspnea": boolean, "Fatigue": boolean, "Fever or chills": boolean, "Headache": boolean, "Loss of taste or smell": boolean, "Muscle or body aches": boolean, "Nausea or vomiting": boolean, "Sore throat": boolean}
>
> Never explain yourself, and only reply with JSON.

Context:

> <VAR_PREVIOUS_JSON_OUTPUT>

VerboseJSONValidation Strategy

Instruction:

You are a helpful assistant identifying symptoms from emergency department notes that could relate to infectious respiratory diseases.

Output positively documented symptoms, looking out specifically for the following: Congestion or runny nose, Cough, Diarrhea, Dyspnea, Fatigue, Fever or chills, Headache, Loss of taste or smell, Muscle or body aches, Nausea or vomiting, Sore throat.

Symptoms only need to be positively mentioned once to be included.

Do not mention symptoms that are not present in the note.

Follow these rules:

Rule (1): Symptoms must be positively documented and relevant to the presenting illness or reason for visit.

Rule (2): Medical section headings must be specific to the present emergency department encounter.

Include positive symptoms from these medical section headings: "Chief Complaint", "History of Present Illness", "HPI", "Review of Systems", "Physical Exam", "Vital Signs", "Assessment and Plan", "Medical Decision Making".

Exclude all symptoms from these medical section headings: "Past Medical History", "PMHFamily History", "FHX", "Social History", "SHX", "Medications", "Allergies", "Imaging", "Diagnostic Study".

Rule (3): Positive symptom mentions must be a definite medical synonym.

Include positive mentions of these medical terms: "anosmia", "loss of taste", "loss of smell", "rhinorrhea", "congestion", "discharge", "nose is dripping", "runny nose", "stuffy nose", "cough", "tussive or post-tussive", "cough is unproductive", "productive cough", "dry cough", "wet cough", "producing sputum", "diarrhea", "watery stool", "fatigue", "tired", "exhausted", "weary", "malaise", "feeling generally unwell", "fever", "pyrexia", "chills", "temperature greater than or equal 100.4 Fahrenheit or 38 celsius", "Temperature >= 100.4F", "Temperature >= 38C", "headache", "HA", "migraine", "cephalgia", "head pain", "muscle or body aches", "muscle aches", "generalized aches and pains", "body aches", "myalgias", "myoneuralgia", "soreness", "generalized aches and pains", "nausea or vomiting", "Nausea", "vomiting", "emesis", "throwing up", "queasy", "regurgitated", "shortness of breath", "difficulty breathing", "SOB", "Dyspnea", "breathing is short", "increased breathing", "labored breathing", "distressed breathing", "sore throat", "throat pain", "pharyngeal pain", "pharyngitis", "odynophagia".

Exclude these symptoms: "injury related to loss of taste or smell", "wheezing", "crackles", "croup", "loose stool", "bloody stool", "looked ill", "afebrile", "felt warm", "headache due to injury", "localized pain", "injury", "abdominal pain", "lower back pain", "gastritis", "gastroparesis", "BiPAP", "CPAP", "oxygen need", "streptococcus", "dysphagia", "hoarseness", "red throat".

Your reply must be parsable as JSON.

Format your response using only the following JSON schema: {"Congestion or runny nose": boolean, "Cough": boolean, "Diarrhea": boolean, "Dyspnea": boolean, "Fatigue": boolean, "Fever or chills": boolean, "Headache": boolean, "Loss of taste or smell": boolean, "Muscle or body aches": boolean, "Nausea or vomiting": boolean, "Sore throat": boolean}. Each JSON key should correspond to a symptom, and each value should be true if that symptom is indicated in the clinical note; false otherwise.

Never explain yourself, and only reply with JSON.

Context:

<VAR_CLINICAL_NOTE>

===SECOND REQUEST===

Instruction:

If the following text is parseable, valid JSON, do nothing and return the text as is.

Otherwise, remove all non-JSON information. Then format the text so that it's valid, parsable JSON that conforms to the following JSON schema: {"Congestion or runny nose": boolean, "Cough": boolean, "Diarrhea": boolean, "Dyspnea": boolean, "Fatigue": boolean, "Fever or chills": boolean, "Headache": boolean, "Loss of taste or smell": boolean, "Muscle or body aches": boolean, "Nausea or vomiting": boolean, "Sore throat": boolean}

Never explain yourself, and only reply with JSON.

Context:

<VAR_PREVIOUS_JSON_OUTPUT>
